# Long-distance movement dynamics shape host microbiome richness and turnover

**DOI:** 10.1093/femsec/fiae089

**Published:** 2024-06-10

**Authors:** William S Pearman, Grant A Duffy, Neil J Gemmell, Sergio E Morales, Ceridwen I Fraser

**Affiliations:** Department of Marine Science, University of Otago, 310 Castle St, Dunedin 9016, New Zealand; Department of Anatomy, School of Biomedical Sciences, University of Otago, 270 Great King Street, Dunedin 9016, New Zealand; Department of Microbiology and Immunology, School of Biomedical Sciences, University of Otago, 720 Cumberland St, Dunedin 9016, New Zealand; Department of Marine Science, University of Otago, 310 Castle St, Dunedin 9016, New Zealand; Department of Anatomy, School of Biomedical Sciences, University of Otago, 270 Great King Street, Dunedin 9016, New Zealand; Department of Microbiology and Immunology, School of Biomedical Sciences, University of Otago, 720 Cumberland St, Dunedin 9016, New Zealand; Department of Marine Science, University of Otago, 310 Castle St, Dunedin 9016, New Zealand

**Keywords:** host, long-distance movement dynamics, microbiome, richness, turnover

## Abstract

Host-associated microbial communities are shaped by host migratory movements. These movements can have contrasting impacts on microbiota, and understanding such patterns can provide insight into the ecological processes that contribute to community diversity. Furthermore, long-distance movements to new environments are anticipated to occur with increasing frequency due to host distribution shifts resulting from climate change. Understanding how hosts transport their microbiota with them could be of importance when examining biological invasions. Although microbial community shifts are well-documented, the underlying mechanisms that lead to the restructuring of these communities remain relatively unexplored. Using literature and ecological simulations, we develop a framework to elucidate the major factors that lead to community change. We group host movements into two types—regular (repeated/cyclical migratory movements, as found in many birds and mammals) and irregular (stochastic/infrequent movements that do not occur on a cyclical basis, as found in many insects and plants). Ecological simulations and prior research suggest that movement type and frequency, alongside environmental exposure (e.g. internal/external microbiota) are key considerations for understanding movement-associated community changes. From our framework, we derive a series of testable hypotheses, and suggest means to test them, to facilitate future research into host movement and microbial community dynamics.

## Teaser

To date we have lacked a framework for understanding microbiome–movement dynamics with regards to host dispersal, we use both ecological simulations and literature to develop this framework—providing a testable framework for future studies.

## Introduction

Differences in host habitat often lead to profound differences in the composition of a host’s microbiome (Kim et al. 2021, Fackelmann et al. 2023). These changes in microbial communities can result from exposure to different microbes (thus increased competition between incumbent and invading microbes), differences in the environmental habitat of the host (e.g. changes to temperature or light exposure), or differences in the host as a habitat (changes in the host that alter it as a habitat for microbes, e.g. stress or changes in diet). On a local scale, such as movement within the ‘home-range’ of a host (Wolf et al. 2021), there is a notable influence of the environment/habitat on the microbiota alongside host-specific characteristics such as sex (Corl et al. 2020). However, in organisms which undertake periodic or occasional long-distance movements (e.g. migrations), there is frequently an associated major change in the environment, which may significantly affect the microbiota—thus we focus here on long-distance movements rather than short-distance (i.e. home-range) movements. Given that ∼1800 birds (Rolland et al. 2014), ∼900 fish (Delgado and Ruzzante 2020), and numerous mammals and insects (Fudickar et al. 2021) are considered migratory (not to mention the frequent occurrence of long-distance dispersal events; Gillespie et al. 2012), there is both a need and opportunity to understand how host-associated microbial communities are shaped by host movements. In this perspective piece, we explore the relationship between host movement (on a broad geographic scale) and microbiota composition/diversity. We then provide an ecological framework, which can be used to develop testable hypotheses regarding host–microbe relationships during the host-movements.

For simplicity, we focus on two types of long-distance movement which we term ‘regular’ and ‘irregular’ movement. We use the term regular to describe repeated and often cyclical movements between geographically distinct locations (e.g. organisms migrating between breeding and feeding grounds; Dingle and Drake 2007, Dingle 2014a). Irregular dispersal, on the other hand, refers to movement of a host to a new and geographically distinct environment where such movements often occur stochastically and without accompanied physiological preparation (e.g. hydrochorous seed dispersal, or *Daphnia* on birds; Green et al. 2008), though this category also extends to more-directed one-time movement events (e.g. eels migrating to spawning grounds at the end of their life; Dingle 2014a,b). There is broad overlap between these terms and terms such as ‘migration’ or ‘dispersal’ (Fryxell et al. 2011), but migration and dispersal carry nuances that limit their clarity for our purpose. For example, migratory movements can occur singularly or repeatedly, each with vastly different effects on the microbiota. In such instances, a singular migration event may be more similar to a stochastic long-distance dispersal event (e.g. migrations for reproduction in semelparous organisms; Dingle 2014a,b). Furthermore, the term dispersal is often reserved to describe movement for the purposes of reproduction (i.e. movement that results in gene flow; Bonte and Dahirel (2017), though see Vickers et al. (2023) for arguments in favour of nonreproductive dispersal), but irregular long-distance movements may occur for myriad other reasons. Finally, ‘migration’ in a stricter sense may be limited to bidirectional, and often seasonal movements, while dispersal could be defined as one-way movement away from a source population. Although these definitions are useful, they favour binary states that do not fit well with the known complexities of many organisms and their microbiomes—we therefore use ‘regular’ and ‘irregular’ dispersal to compare processes.

Regularly moving organisms comprise almost entirely animals, while irregular movement is typical of a broad array of plants and spawning animals and can also include adult hosts (e.g. kelp rafts) or even entire marine communities (e.g. biofouled flotsam; Oberbeckmann et al. 2016). However, we suggest that many commonalities can be found within each category based on shared ecology rather than the taxonomic identity of the host (Moraitou et al. 2022). Although there are caveats within these groups (e.g. regular dispersers are necessarily active dispersers, but can also undertake irregular dispersal), we employ these terms principally because we suggest that the primary factor that shapes microbiota responses to movement is the large changes in habitat resulting from the type of movement. Consequently, the average habitat of a regular disperser is thus drastically different to the average habitat of an irregular disperser—even where such dispersers belong to the same population (Risely et al. 2018, Turjeman et al. 2020). In essence, we suggest that it is not host identity or type that is the key determinant of movement-associated shifts, but it is the type of movement.

Both regular and irregular forms of movement are distinct from localized movements where, although the microbiota may be variable, the environmental medium through which movements occur remains more hospitable relative to the migratory movement medium. For example, although the microbiota may be correlated with an organism’s ‘home range’ or environment (Wolf et al. 2021), the differences in ecological conditions (e.g. food type, temperature, and salinity) are small, relative to those found across a migratory journey (Lewis et al. 2017). Indeed, migratory movements are, beyond their simple geographic change, also examples of environmental change (Tøttrup et al. 2008, Eisaguirre et al. 2019). Environmental changes are well-established in shaping the microbiota (Rothschild et al. 2018, Casto-Rebollo et al. 2023), however, the extent of changes within a migratory movement may far exceed the degree of change observed within a singular environment.

Although long-distance movement is commonplace among plants and animals (Gillespie et al. 2012, Rolland et al. 2014, Delgado and Ruzzante 2020, Fudickar et al. 2021), we know little about how established microbiota are affected when their hosts undergo long distance movements. Yet, these movements are somewhat analogous to a perturbation or community disturbance, which have been frequently linked to changes in the microbiota (Sommer et al. 2017, Santillan et al. 2019). For some host organisms, these disturbances principally alter the microbiota through changes in the dominance of core and noncore taxa (Pearman 2023), while for others core taxa remain persistent throughout the migratory process (Wu et al. 2018, Víquez-R et al. 2021). These data, alongside emerging research, highlight the profound impact (such as high species turnover/beta-diversity, or substantial restructuring) of long-distance movements on established microbiota (Bierlich et al. 2018, Skeen et al. 2021) in response to new environments and exposure to novel microbes. Nevertheless, current studies largely represent discrete timepoints within continuous processes (because e.g. sampling at stopover sites within a migration is straightforward, while sampling birds in flight is much less so).

Identifying how long-distance movement events impact host-associated microbial communities will be key to understanding fundamental ecological processes in highly dynamic environments and predicting how species on the move may perform in their new environments. Recent work by Grond et al. (2023) revealed that the microbiome plays a significant role during shorebird migration through influencing weight gain by synthesis of fatty acids. Such processes in shorebirds are also observed during summer weight gain in hibernating bears, highlighting the influence of the microbiome in shaping core survival processes of multiple host species (Sommer et al. 2016). These data thus support the hypothesis that the migratory process is a disturbance, albeit an extreme one, leading to changes in the selective pressures on a community, which in turn result in compositional shifts in the microbiota (Schmid et al. 2023). Nonetheless, as stated above, with successive migratory events the ‘average’ habitat becomes closer to the migratory habitat—which may result in movement-associated changes falling within the intermediate disturbance hypothesis.

Changes in the microbiota that may result from irregular long-distance movements are rarely studied, likely due to the inherent difficulties in sampling organisms, which disperse stochastically and across extremely varied distances (Gillespie et al. 2012). However, we suggest that some insights into these processes may be drawn from translocations—which are (from a host perspective) an unpredictable change in habitat (although there is an absence of a migratory environment in the same sense as a migrating organism). Where possible we draw on the scant literature examining irregular dispersal and attempt to fill in the blanks with simulations and translocation based literature (e.g. Webster et al. 2020).

Global anthropogenic change is leading to major range shifts for a swathe of organisms—resulting from a melting pot of species invasions (e.g. through competitive exclusion; Mooney and Cleland 2001), habitat change/destruction (Taylor and Stutchbury 2016), and shifting dispersal pathways (e.g. ocean currents) (García Molinos et al. 2022). Such range shifts are underpinned by dispersal of hosts, and thus understanding host–microbiome dynamics with regards to irregular dispersal is essential. Indeed, given the already established role of the microbiome in invasion success of a diversity of taxa (e.g. codispersed microbes are not beneficial in legumes, but are in some algae; Simonsen et al. 2017, Bonthond et al. 2021) there are practical uses for understanding the microbiota of dispersing organisms. Specifically, understanding the effects that the host dispersal process has on microbiota structure may aid in identifying which taxa may be of greater invasion concern, especially as these invasion events occur with greater frequency.

Independent of host movements, microbes continually relocate and disperse within and among hosts and environments (Burns et al. 2017, Stothart et al. 2021, Custer et al. 2022). Here, we focus on the effects of long-distance host movements on associated microbial communities and thus, except where explicitly stated, movement and dispersal refers to that of the host rather than of microbes (Dingle and Drake 2007). Nevertheless, we acknowledge that microbe movement will be affected by, and interact with, host movement and associated processes (Bo and Kohl 2021).

## Challenges and emerging trends

Studying host microbiota during migration often requires navigating geo-political barriers and sampling organisms underway (e.g. flying birds). As a result, most studies focus on co-occurring resident/migrant individuals (Risely et al. 2017, 2018, Bierlich et al. 2018, Turjeman et al. 2020), or examine start, stopover, and end points of migratory movements (Skeen et al. 2021, Thie et al. 2022, Grond et al. 2023). Nevertheless, some patterns appear to be emerging. In migratory birds, microbiota richness decreases immediately following long-distance movement and steadily recovers during residency (Risely et al. 2018, Skeen et al. 2021). In other bird species, microbiota diversity has been observed to correlate negatively with migratory distance when sampled at a stopover site (Thie et al. 2022); such results are found across a range of migratory organisms (Bierlich et al. 2018, Skeen et al. 2021). When the results of Bierlich et al. (2018) and Vendl et al. (2019, 2020) are considered together, an interesting picture emerges that aligns with patterns observed in birds; the migratory process is associated with a decline in species richness, but recovers following migration. Meanwhile, despite the relative paucity of research on microbiota of more stochastic host movements, rafts of the brown alga *Sargassum* in the Northern Hemisphere show higher microbial turnover than coastal or attached hosts (Michotey et al. 2020), a pattern mirrored in rafts of brown algae in the Southern Hemisphere (Pearman 2023).

Thus far, we have focused on the microbiota in a broader sense, but whether such a community is internal or external to the host will also make a difference to the influence of long-distance movements. In an artificial movement setting (translocations), equine caecal microbiota declined in species richness during travel (Perry et al. 2018), whereas the bovine nasopharyngeal microbiota increased in richness (Chai et al. 2022). Such shifts in microbiota structure may result from differences in exposure levels during movement, prior exposure to movement environments, or differential effects of movement-stressors on the microbiota (e.g. hosts adapted to migration versus those not adapted).

The frequency and nature of host movements can alter host exposure levels and metabolic requirements, and impact host-mediated processes on the community. Distinctions between the impacts of regular and irregular movements on the microbiota may arise due to the evolutionary significance and adaptation of many organisms to migratory movements (e.g. immune function is affected by migration; Eikenaar et al. 2020; and immunogenetic variation is in turn linked to microbiota structure; Green et al. 2008). Meanwhile irregularly moving organisms may lack such adaptations (or even be aided by losing their original microbiota; Simonsen et al. 2017). In essence, there are differences in the microbiota of organisms, which migrate repeatedly throughout their life and those of organisms which only migrate once. If long-distance movements are frequent or repeated, their regularity means that hosts could develop adaptations to long-distance movement (Dingle and Drake 2007, Turjeman et al. 2020), which may buffer the microbiota from external stressors. These regular host movements are fairly predictable and thus straightforward to study, and indeed considerable research has been carried out on the microbiota of migratory organisms (e.g; Kreisinger et al. 2015, Lewis et al. 2017, Bierlich et al. 2018, Grond et al. 2018, Risely et al. 2018, Wu et al. 2018, Vendl et al. 2019, Turjeman et al. 2020, Skeen et al. 2021, Obrochta et al. 2022). In contrast, the often-unpredictable nature of irregular host movement is reflected in relatively few studies on microbiota changes with such events.

## Insight from simulations

Although empirical studies are sparse and moving organisms are difficult to study, simulations allow testing and development of hypotheses. When informed by empirical data for parameterization and ground truthing, such simulations can enhance our understanding of the implications, processes, and patterns involved in microbiota changes with host movement. Depending on the scale of interest (and availability of information), models can be parameterized to reflect site-specific conditions or a whole-organism average that aims to balance model complexity, fit, and ‘usefulness’. To this end, we employed demonstration models exploring microbiota–movement dynamics, these models approximate basic community dynamics and assembly/turnover over sequential time steps/generations and can incorporate variation in environmental selection, and its interaction with species traits, and changes in exposure to environmental species pools through time (Box 1; https://github.com/wpearman1996/Dispersal_simulations).

Box 1. **Overview of simulation methods.**

Modelling was performed using the *ecolottery* package (Munoz et al. 2018) implemented in R Statistical Software (R Core Team 2022). Models comprised communities of simulated species representing a generic host microbiota. We initialized communities using species lists derived from water samples collected either as part of a coastal macroalgal sampling effort (Pearman et al. 2023), or off-shore sampling from the Munida Microbial Observatory Time Series dataset (Lockwood et al. 2022). Each species was assigned a trait drawn on a univariate scale. Individual fitness in each generation was calculated based on the distance between traits (i.e. the individuals optimal environment) and the current environment (a location on the same univariate scale that varied to replicate host movement) and the amount of trait overlap with other individuals (i.e. competition). At each time step all individuals had a chance of dying based on their relative fitness, with removed individuals immediately replaced by individuals of surviving species or immigrants from an environmental species pool.

The environment was defined as the complete set of selective pressures imposed on a community from both biotic and abiotic sources, rather than being strictly abiotic—taking the view that from a microbial perspective, the environment encompasses the host. This approach means that the selection imposed on community is univariate and analogous to the first principal component of all selective pressures. The community of simulated species transitioned from their ‘origin’ environment, through a ‘movement’ environment, and into a ‘destination’ environment. Each environment (origin, movement, or destination) exerted selection on this scale via a Gaussian function centred around the selection parameter of each environment with a sigma value of 0.05. Assuming relative similarity between origin and destination environments (e.g. coastal regions separated by open ocean), selection was centred around 0.3 for the origin and 0.4 for the destination. Meanwhile, selection in the movement environment (e.g. the open ocean itself) was centred around 0.7 to reflect that movement often occurs across environments that are dissimilar to either the origin or destination environments. To emulate natural environmental gradients, transitions from one environment to the next were gradual. Therefore, selection on the first 10% and last 10% of generations in the Movement period linearly increased or decreased to/from 0.7 to smooth the transition from/to origin or destination conditions. For a detailed description of simulation methods, including code, see the Supplementary Material.

We broadly defined the microbiota as any delineated microbial community associated with a host, with membership being any microbe with a nonzero abundance. This definition thus includes transient microbes within the community, though many such microbes occur at such low abundances that they are lost rapidly or have no significant effect on the community (Supplementary Figures). Although we did not define a core microbiome across all simulations (as each host is modelled independently rather than within a host population context), we divided the community into abundant/rare taxa based on an average abundance across the simulations (abundant taxa were those with an average abundance >0.1%). Host organisms comprise a broad array of microbiota (e.g. skin, gastrointestinal, and oral), each exposed to differing environments and selection pressures. Therefore, to understand the effect of differing exposure levels (i.e. understanding internal/external communities), we varied the species richness of the environmental community. From our simulations, we then analysed species richness, dissimilarity (Bray–Curtis), and functional diversity (based on microbial trait values).

**Figure 1.**
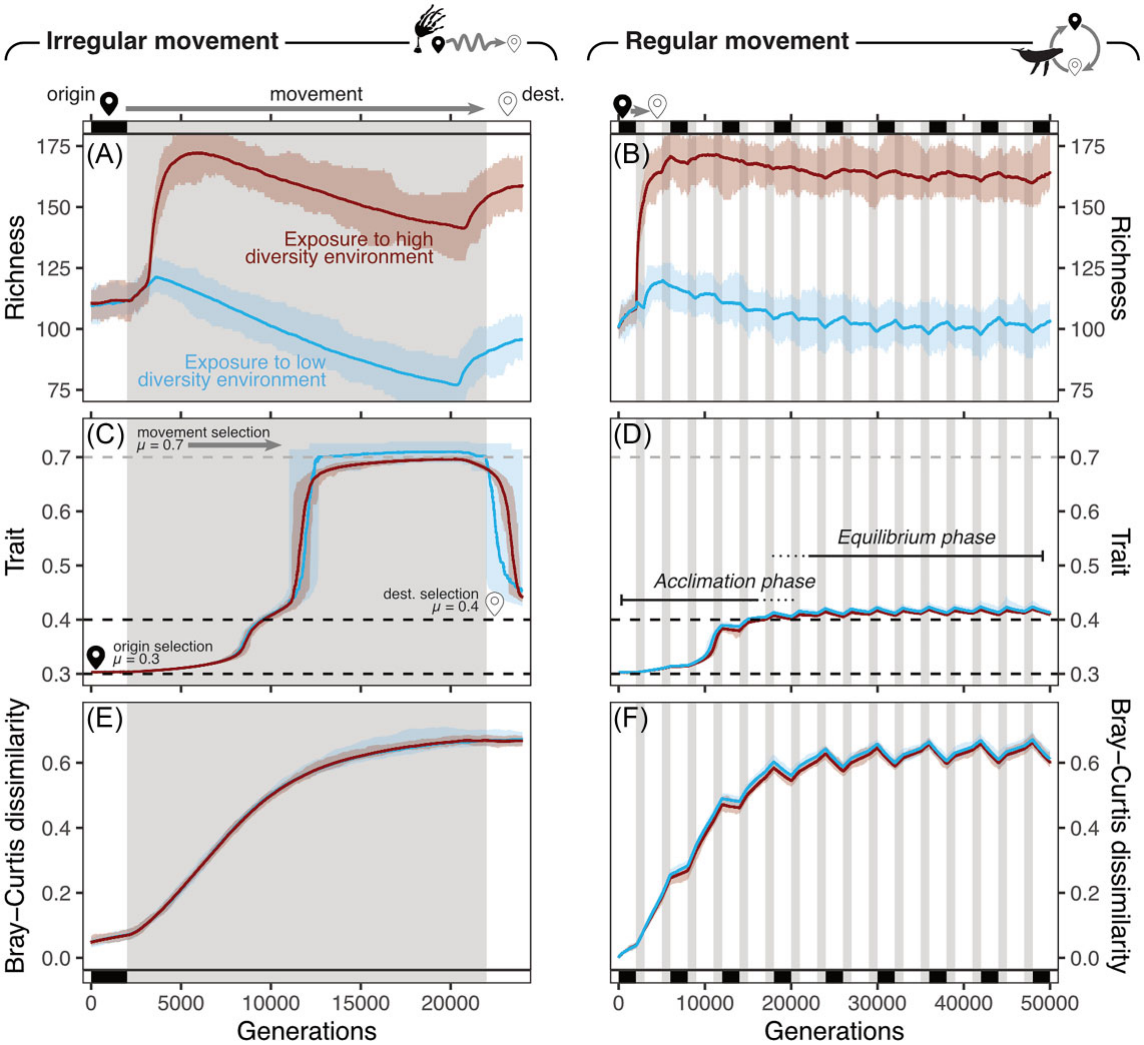
Influence of movement on simulated microbiota species richness, functional diversity (as traits on a univariate scale), and dissimilarity. Grey shading indicates movement periods. Horizontal axes represent time in generations of simulated microbes. Left-hand panels (A, C, and E) show irregular host movement, while right-hand panels (B, D, and F) show regular host movement. Line colour reflects the relative richness of the environmental pool of microbes during movement (blue = low diversity pool and red = high diversity pool).

Despite their broad parameterization, our simulations produce results that are analogous to those observed in real plant and animal communities (e.g. Bierlich et al. 2018, Risely et al. 2018, Webster et al. 2020, Skeen et al. 2021, Chai et al. 2022), suggesting that even simple models can help explain ecological processes. Our simulations demonstrated that long-distance movement influences the membership and relative abundance of host microbiota (Fig. 1A and B; Supplementary Figs 2–4). Microbiota, which underwent irregular movement experienced a shift towards a community dominated by microbes with optimal fitness in the movement environment (Fig. 1C). Conversely, microbiota undergoing regular movement, such as during cyclical migrations, were dominated by microbes with an intermediate fitness across all movement stages (Fig. 1D), rather than optimal fitness during one stage and subpar fitness in all other stages, resulting in relatively consistent patterns in richness and community structure. Host movement could, therefore alter microbiome functional diversity or structure in such cases, as has been observed in migratory birds (Grond et al. 2023). Irregular movements also resulted in a compositional shift in community structure away from the initial community (Fig. 1E), while for regular movements we found that, following the acclimation phase, dissimilarity to the initial community was dependent on whether a community was in the origin or destination location (Fig. 1F). This finding perhaps results from reaching a point of maximum dissimilarity (after accounting for cosmopolitan microbes), and transient changes in microbiota structure in the equilibrated phase result from loss/acquisition of microbes in the origin community, as our simulations do not consider long-term changes in diversity of the environmental pools.

Simulations under a scenario of regular migration resulted in an initial increase in species richness (Fig. 1B), after which richness tended to exhibit a marginal decline during the migratory period—following patterns anticipated under the intermediate disturbance (Connell 1978) or intermediate stochasticity hypotheses (Santillan et al. 2019). These simulated data, alongside observations from the skin microbiota of humpback whales (Bierlich et al. 2018) and faecal microbiota of Kirtland’s warblers (Skeen et al. 2021), paint an intriguing picture of the effects of migration on microbiota structure in regularly migrating organisms. A migratory organism’s first few migrations can be considered analogous to irregular movement events, and can be grouped together as an ‘acclimation’ phase (Fig. 1D), where communities are becoming acclimated to movement events. The repeating nature of regular movement then leads to an equilibration of the microbiome where frequent movement is essentially the ‘new normal’ and the turnover or change in the community over time has begun to stabilize, resembling the constant low levels of turnover found in previous studies (Moya and Ferrer 2016, Priya and Blekhman 2019). Although such stabilization may be expected throughout the development of even nonmigrating organisms (Grond et al. 2018, Roswall et al. 2021), we suggest that the migration-induced disruption to microbial communities is a key determinant of the assembly of a migration-acclimated microbiota found in/on regularly moving hosts.

Microbiota of hosts that move irregularly did not exhibit the same stabilization (Fig. 1A). Thus movement, if it occurs only singularly or for the first instance (e.g. natal dispersal in salmon, or juvenile migratory birds), is comparable to an instance of irregular movement with regards to microbiota structure even if such an organism ultimately will undergo regular movement (transitioning to the equilibrium phase of regular movement). The phases of migration–microbiota equilibration (acclimation/equilibration; Fig. 1D) and changes in diversity in response to migration are also largely shaped by system specific factors (e.g. internal/external, or migrating/nonmigrating). For example, migratory individuals of the same host species, cohabiting the same location, can have distinct microbiota to those of nonmigratory counterparts (Risely et al. 2018, Turjeman et al. 2020), and in a similar way an internal microbiota may be affected differently by migration than an external microbiota.

Increases in richness observed during the acclimation phase are proportional to the richness of the environmental pool to which the microbiome is exposed (i.e. the more potential colonizers, the greater the increase in richness) (Fig. 1A and B). This relationship may explain why travel leads to decreases in richness in gut microbiota (Perry et al. 2018, Obrochta et al. 2022), while nasopharyngeal microbiota increase in richness (Chai et al. 2022). There are two principle reasons why exposure may affect the microbiota of migrating organisms. First, internal communities are perhaps more insulated against extreme environmental fluctuations (e.g. variations in light, temperature, or UV exposure) and thus internal cavities may represent a more stable environment relative to the environment experienced by a skin community (Costello et al. 2009). Secondly, internal cavities are under reduced colonization or invasion pressure relative to a skin microbiota; potential colonizers of internal cavities must be introduced through breathing or food consumption compared to a skin microbiota, which is exposed to the entire ‘microbial soup’ of the environment (Grönroos et al. 2019, Harrison et al. 2019, Vila et al. 2019, Berggren et al. 2023, Santos et al. 2023). Internal cavities of migratory organisms may be under even less colonization pressure during migration due to fasting or altered feeding regimes, which may reduce exposure to new organisms during the migratory process (Klinner et al. 2020).

A comparison of skin and faecal microbiota of wild salmon pre- and post-translocation into a natural environment also supports the hypothesis that exposure can mediate microbiota diversity (Webster et al. 2020). These differences in exposure may explain why internal microbiota frequently decline in richness, while external communities increase in richness (Perry et al. 2018, Webster et al. 2020, Chai et al. 2022), during movement. Such differences may be amplified during migratory events where changing selective pressures as a result of physiological changes may compound with reduced microbial intake (due to altered feeding regimes, or fasting; Nichols et al. 2022), leading to an overall decline in richness. Although we suggest that physiological changes primarily co-occur with regular dispersal, they can also occur in instances of irregular movements (e.g. reproductive migration of eels; Dingle 2014b), which may impose an additional selective pressure.

## The importance of repeated sampling

Our simulations provide continuous data across all generations of the modelled microbiota (Fig. 1), something which is rarely available when collecting data empirically (Jones et al. 2023, Fountain-Jones et al. 2024). Nevertheless, repeated measures of the same individual can provide invaluable insights into drivers of microbial community assembly (e.g. environment, host genetics, and so on; Grieneisen et al. 2023). Repeated snapshot sampling of migrating organisms has already provided core insights into community assembly rules (Skeen et al. 2021), and sampling of meerkats (Risely et al. 2022) and primates within their home-ranges (Björk et al. 2022) has highlighted the variable nature of the microbiota over time, as well the importance of individual identity. Quantifying this on a broader scale and with increasing frequency in migrating organisms would provide an ideal means to quantify the role of environmental distances in shaping community structure relative to the role of the host. Although such questions are of interest, a framework for developing hypotheses around microbiota–migration interactions is presently absent.

## A framework for microbiome interactions with long-distance movement

**Figure 2.**
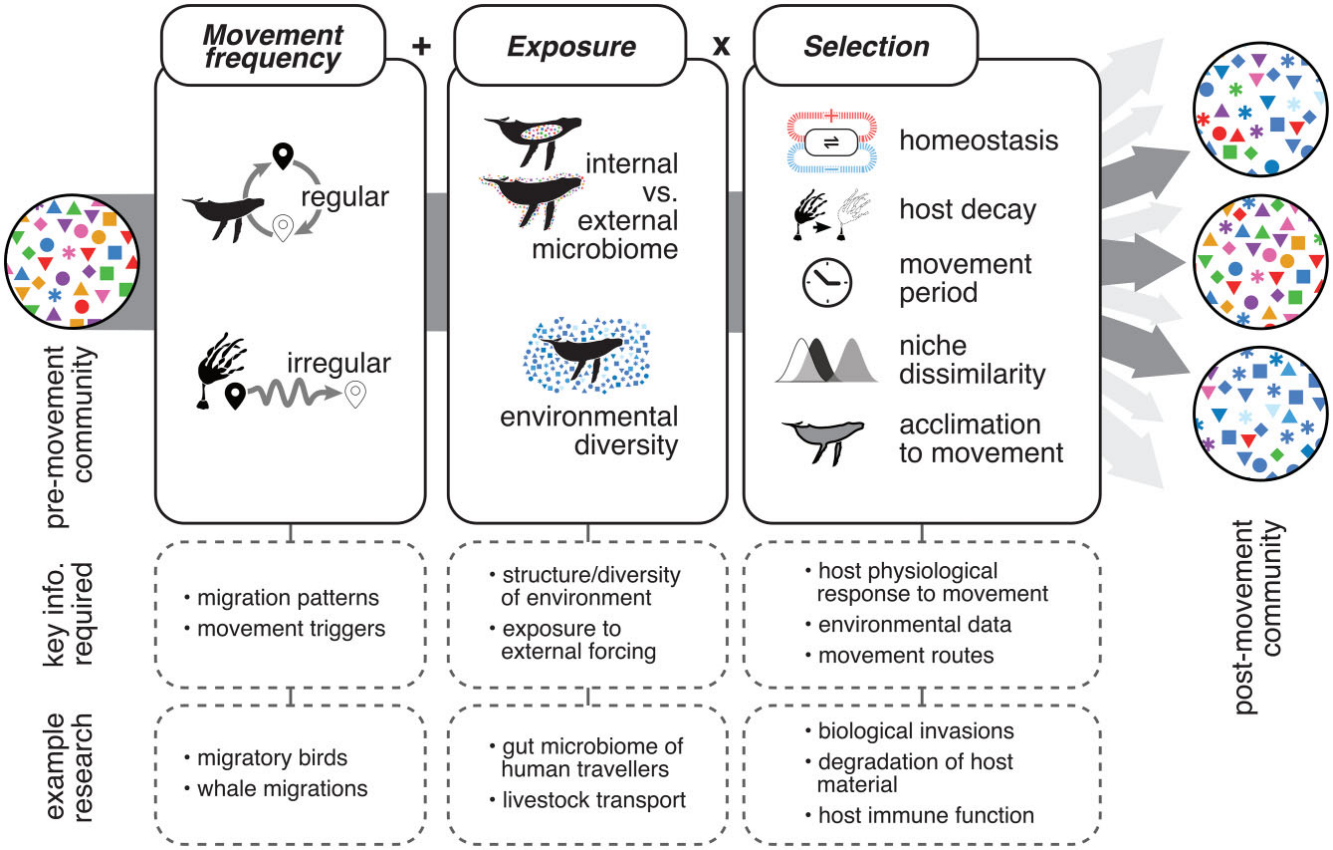
Framework for the study, explanation, and prediction of movement-driven changes to microbiota structure. We propose that postmovement communities are most influenced by the form of movement (i.e. regular versus irregular) while exposure to new microbes in the movement environment and selective processes, such as host homeostasis, which may promote resistance to change or host decomposition which may accelerate change, interact to add to, and/or modulate movement frequency effects.

A conceptual framework could be beneficial for focussing future effort, addressing existing shortfalls, and enhancing understanding of microbiota movement dynamics and contrasting patterns of regularly and irregularly moving organisms. Such a framework enables development of testable hypotheses and questions and allow identification of commonalities across a range of complex systems. We suggest that microbiota diversity (richness and turnover) and function in dispersing organisms are best explained by three groups of interacting factors: movement frequency (e.g. regular/irregular; Fig. 2), exposure (both exposure to the environment, and exposure to other microbes—e.g. internal and internal communities), and selection (imposed by type of movement and associated physiological stress).

### Dispersal frequency

We expect frequent long-distance movement to lead to a movement-equilibrated community as the microbiota respond to less-stable environments, and thus the response to movement decreases in magnitude with increasing frequency of movement. In essence, movement through multiple environments promotes selection for microbes, which can persist through all environments, and thus repeated movements will stabilize on a ‘core’ community in the ‘equilibrium phase’ with reduced turnover relative to ‘acclimation’ phase. The corollary of this hypothesis is that hosts, which rarely undergo long-distance movement will have extremely movement-affected microbiota, which could explain why the microbiota of some migrating whales and birds show relatively little change in community structure when compared to irregularly dispersing organisms (Bierlich et al. 2018, Skeen et al. 2021).

### Exposure

As discussed previously, exposure to external microbes strongly influences changes in community richness (Fig. 1). Our simulations suggested that the primary determinant of microbiota movement responses in the equilibrium phase was the richness of the environmental community (Fig. 1), because greater exposure to the environmental pool of microbes increases the potential for colonization. While not incorporated into our simulations, colonization order and priority effects also promote persistence of incumbent microbes (Sprockett et al. 2018), which may be another reason why many microbiota typically have higher richness following movement. We expect the same principles to apply across tissue types and compartments, with movement-driven microbiota differences arising from different contributions of exposure and selection across tissues/compartments. For example, internal compartments may have very low environmental exposure, resulting in them being shielded from both the environment and potential colonizers (Brown et al. 2020, Zhang et al. 2022) and may experience relatively little movement-prompted community change compared to skin microbiota, which have much higher exposure (Webster et al. 2020).

### Selection

Selection is another determinant of changes in microbiota richness in response to movement, with stronger selection leading to smaller increases in richness (Supplementary Fig. 2). Changes in the microbiota as a result of frequent long-distance movements are underpinned by selection, as frequent movement will select for communities that can withstand that movement (i.e. a ‘core’ microbiome; Risely 2020). However, regardless of movement frequency, selection will shape the microbiota in some way, such as when a host actively regulates their microbiota, potentially promoting resistance to change (Arnault et al. 2022) or alternatively, when such regulation is disrupted leading to decline in the selective processes (Naylor et al. 2017). Fasting and fat loading are both frequently observed behaviours associated with long-distance migrations, and these in turn impose additional selective pressures on the microbiota (Risely et al. 2018, Thie et al. 2022, Grond et al. 2023). Conversely, decomposition and deterioration affects microbiota structure (Preiswerk et al. 2018), and thus hosts which undergo degradation during long-distance movements may experience selection towards degrading/decomposing microbes, leading to greater than expected changes in microbiota structure (Pearman 2023).

Movement events also lead to the exposure of a host to a broad range of environments (Tøttrup et al. 2008), each of which may represent both a source of new microbes and a source of selection (Thie et al. 2022). In turn, movements may expose hosts to a new suite of diseases and pathogens, which can amplify changes in the microbiome (Zhang et al. 2021). Although the relationship between long-distance movements and disease has not been comprehensively explored, some studies have proposed the use of microbiome surveillance as a tool to detect pathogens such as *Salmonella* (Choi et al. 2021). Indeed, given the role of bird migration in transmission of avian influenza (Sun et al. 2022) and the recently determined importance of stopover sites in the establishment of avian pathogens in migrating birds (Włodarczyk et al. 2024), there is a clear need to understand the relationship between microbiome composition, pathogens, and long-distance movements.

Changes in selection can arise from a variety of sources, and the magnitude of the changes in the microbiota likely arise from the magnitude of selection. One principal source of selection may be the environmental dissimilarity between the origin and movement environments. For example, flamingos largely reside in hypersaline environments, but migrate through hypo- or meso-saline environments (Amat et al. 2005). Such extreme differences likely contribute to changes in the community in the same way that environmental exposure can shape the microbiota. A ‘migration-equilibrated’ microbiota could also feasibly occur via means of dormancy, where microbes which are ill-suited to the migratory medium enter a state of a dormancy until exposed to more favourable conditions in the origin/destination environments, thus temporarily bypassing selection that may be acting on nondormant members of the community. For example, the persistence and presence of halophilic microbes in migrating flamingos (Yim et al. 2015, Kemp et al. 2018, Gillingham et al. 2019) may be aided by dormancy. Although dormancy has been frequently described in nonhost-associated microbiota (Jones and Lennon 2010, Locey et al. 2020, Sorensen and Shade 2020), to our knowledge no studies have yet explored dormancy in microbiota associated with a host.

### Considerations and application

While our framework (Fig. 2) is primarily relevant to pre-established communities, reduced vertical transmission (acquisition of microbes from parents) may be advantageous in fluctuating environments such as those resulting from and/or experienced during, long-distance movement (Bruijning et al. 2022). We suggest that while vertically acquired microbes may have an initial advantage over horizontally acquired microbes (microbes acquired from the environment), this can be explained by either priority effects (Sprockett et al. 2018) or selection (Zeng et al. 2017). In the case of the former, there is no reason to expect that their presence due to vertical transmission has any bearing on their response to movement. However, in the case of vertical transmission of positively host-selected microbes, we would anticipate that these microbes may be more resistant to movement-driven changes and thus under positive selection. These beneficial microbes could be considered holobiotic and may be a source of inertia for resisting movement-prompted changes to the microbiota. Nevertheless, disentangling the contributions of different ecological factors to observed microbiota diversity remains critical, especially given the presence and possible interactions between factors such as priority effects, migration timing, environmental conditions and diversity, and seasonal variability (Zhang et al. 2021).

Although microbial communities are highly dynamic, and have been observed to undergo daily (Zarrinpar et al. 2014, Zhang et al. 2023) or seasonal cycles (Huang et al. 2022, Schmid et al. 2023), there remains strong evidence for priority effects on microbial communities (Martínez et al. 2018, Leopold and Busby 2020). However, the influence of priority effects may be linked to both the degree of disturbance induced by long distance movements (e.g. higher disturbance levels or higher environmental variability may overcome priority effects; Tucker and Fukami 2014), and the time between movement events. In the latter instance, increased microbial generations between movements is likely to be associated with reduced influence of historic movements. All of these nuances are capable of occurring in a ‘neutral’ host system (where the host is neutral, but selection operates within the community), however in systems where the host may actively regulate or maintain a movement-adapted microbiota such priority effects may be of adaptive benefit to the host (Debray et al. 2023).

Our framework can be applied to various host-associated microbiota, noting that different host taxa may emphasize different elements. For example, seed microbiota are likely to be more influenced by environmental conditions (Morales Moreira et al. 2021), while some migratory birds may have greater host-directed mediation of the microbiota (e.g. microbiota–immune system interactions; Bolnick et al. 2014, Leclaire et al. 2019, Fleischer et al. 2022). Therefore, although plants are not migratory in the same sense that many birds are, they still undergo long-distance dispersal and can thus still fit within our framework. Specifically, plants would frequently fall into the acclimation phase (Fig. 2) as the adapted phase is found in those organisms, which undergo repeated movements.

Despite the clear and well-documented influence of host processes (Blekhman et al. 2015, Xiong et al. 2021), there remains a large body of work which points to microbiota being explained by predominantly ecological processes that are independent of the host (i.e. are host agnostic) (Adair and Douglas 2017, Rothschild et al. 2018, Mukherjee et al. 2021, Moraitou et al. 2022). Such host-independent factors are well-represented in the proposed framework (Fig. 2) and, coupled with the knowledge that host–microbe interactions can modify community structure further, this provides a platform from which hypotheses for specific organisms can be developed.

## Key hypotheses and how to test them

Using our framework (Fig. 2), we propose a range of testable hypotheses that could give improved insight into microbiota–movement dynamics, and into the mechanisms through which host microbiota change with host movements (H1–H7). We then outline existing and emerging experimental approaches (A–C) that could be used to test these hypotheses. While we focus here on movement dynamics, long-distance movements are, by their nature, examples of extensive environmental change.

### General hypotheses

H1) Strong host selection (e.g. homeostatic regulation) will lead to less microbiota change as a result of a movement event. Conversely, a lack of host regulation will amplify the effect of movement on microbiota structure.
H2) The ecological processes governing microbiota turnover will shift towards selection [both host- and nonhost imposed; assessed following methods developed by Stegen et al. (2013) and Ning et al. 2020)] following long-distance movements, but the influence of selection will decline with successive movements. This expectation is because sudden environmental changes impose a major environmental filter on a microbiota, but successive movements bring this movement closer to the ‘average’ environment.
H3) Due to priority effects (Sprockett et al. 2018, Sieber et al. 2019), incumbent microbes will be retained throughout the movement period, albeit with decreased abundance, even if they are ill-suited to movement. For example, microbes which are adapted to the origin may be retained through the migratory process, even if they have lower fitness during the migratory process. Such a scenario may arise jointly from priority effects or from dormancy of those microbes during long-distance movements.
H4) We hypothesize that internal microbial communities will respond differently (but not necessarily less dramatically) to migration than external microbiota owing to differences in exposure to environmental microbes.
H5) During long-distance movements, turnover of bacteria is elevated (driven by loss of transient/noncore microbes) and relative abundances might differ, while the composition of core community remains stable.

### Hypotheses specific to regular movement

H6) Hosts undertaking their first migration will experience rapid uptake of environmental microbes and saturation of microbial richness, and will initially show similar patterns in microbiota structure to irregularly dispersing hosts.

### Hypothesis specific to irregular movement

H7) Microbial richness will rapidly saturate during movement, but microbial turnover will continue to increase until the host settles in a new environment.

Across the board, longitudinal sampling of host microbiota over the course of regular and irregular movement events could substantially advance our understanding of how host movement can shape microbiota. Prioritizing repeated sampling of microbiota, to enable study of microbiota successional trajectories and turnover within changing environments, would be a valuable component of such studies. However, recognizing that directly following and sampling dispersing hosts is often infeasible, here we suggest a trifecta of empirical means to test the above hypotheses.

- *Manipulative experiments*: laboratory mesocosms would enable direct control and modification of movement/disturbance frequency, environmental exposure, and selection. Such systems would enable experimental testing of H1-4 and cyclical variation of the controlled environment would enable assessment of the transition from equilibrium to acclimation phases. In essence, these mesocosms would manipulate the environment around the host to mimic the migratory environment (i.e. the environment moves around the host, rather than host moving through the environment). Mesocosms with uniquely barcoded, but otherwise identical, bacteria would prove especially helpful for testing the effects of selection and immigration in isolation, following those experiments conducted by Cira et al. (2018).
- *Field observations*: we suggest that new methods for tracking and opportunistic sampling of dispersing hosts should be explored, with the integration of complementary methodologies an area of particular promise. For irregular movement, opportunistic sampling combined with movement modelling (e.g. particle drift modelling) (Michotey et al. 2020) and genetic inference of origin populations could provide pseudolongitudinal data to test H1, H2, H4, and H5.
- *Host proxies*: sampling proxy ‘hosts’, such as biofilms from marine debris (Oberbeckmann et al. 2016) or vessel hulls, could also be informative. Comparisons of vessels, which undertake frequent and consistent long-distance trips (e.g. commercial fishing boats or cargo ships) to those which undertake intermittent trips (e.g. leisure boats) would enable the assessment of H2–H3 and H5–H7. Such systems would be particularly useful for understanding how the microbiota of simplistic hosts can be shaped by the movement process—providing core insights on how communities shift in response to movement in the complete absence of host regulation. For example, host regulation of the microbiota in such an instance would be simplified down a basic factor of antibiofouling paints—enabling much more direct testing.

## Outlook

Ultimately, greater understanding of microbiota–movement dynamics requires the application of a range of both experimental and observational approaches. We suggest that movement frequency, the level of exposure to environmental microbes, and selection (both host- and environmentally imposed) are the primary drivers that shape how microbiota respond to long-distance movements. The need for such a framework was prompted by a recognition of the importance of, but relative paucity of literature examining, microbiota response to irregular movement. Our framework allows us to derive a range of testable hypotheses regarding microbiota dynamics during host-movements, which we hope will initiate and guide future research directions. Special focus should be given to disentangling the relative contributions of movement frequency, environmental diversity, and selection in shaping the microbiota, and using these to bridge experimental and observational studies. These studies have critical relevance to biosecurity in terms of disease movement and predicting future microbial scenarios in the context of rapid environmental and climatic change.

## Supplementary Material

fiae089_Supplemental_Files
