# Supplementary material for: Long-distance movement dynamics shape host microbiome richness and turnover
Source: FEMS Microbiol Ecol. 2024 Jun 10;100(7):fiae089. doi: 10.1093/femsec/fiae089 (PMC11212666; doi:10.1093/femsec/fiae089)
Supplement: fiae089_Supplemental_Files [file fiae089_supplemental_files.zip › markdown_supp data.pdf]

Pearman *et al.* - migration simulation

2024-03-04

Using the `ecolottery` package to simulate a community migrating from a 'origin' to a 'destination' via a 'movement' environment.

```
require(ecolottery)
require(ggplot2)
```

Load custom functions for random draw migration simulations.

```
source("functions/forward_mod.R") # custom `forward` function to output communities directlyto csv
source("functions/forward_shuffle.R") # custom function to shuffle microbiome amongst multiple hosts di
```

Set up a niche filter function and constant parameters used in all pools/sims. Baseline (i.e. before integrating niche components) probability of an organism dying. Probability of dead inviduals being replaced by immigrants.

```
filt_gaussian <- function(t, sigma, x) exp(-(x-t)^2/(2*sigma^2))

prob_imm <- 0.3  #immigration
prob_death <- 0.05 #death baseline
lim_sim <- TRUE #limit similarity
coeff_limsim <- 1
lim_sim_sigma <- 0.05
nind <- 500 #size of microbiome - keeping small for testing

cohort_size <- NULL # number of co-dispersing hosts - if NULL single host disperses with no 'shuffling'
```

Species pool for the origin environment.

```
origin_start <- read.csv(file = "data/source_start.csv", row.names = 1)
```

Species pool for the movement environment.

```
movement_start<-read.csv(file = "data/dispersal_start.csv", row.names = 1)
```

Species pool for the destination environment.

```
destination_start<-read.csv(file = "data/sink_start.csv", row.names = 1)
```

Add random noise to individual species traits to emulate within-species trait variability.

```
origin_start$trait <- truncnorm::rtruncnorm(1, a = 0, b = 1, mean = origin_start$trait, sd = 0.02)
destination_start$trait <- truncnorm::rtruncnorm(1, a = 0, b = 1, mean = destination_start$trait, sd = 
movement_start$trait <- truncnorm::rtruncnorm(1, a = 0, b = 1, mean = movement_start$trait, sd = 0.02)
```

Parameters for each stage of the simulation.

```
ngen_origin <- 200 #Number of generations in the origin environment
ngen_movement <- 100 #Number of generations in the movement environment
ngen_destination <- 200 #Number of generations in the destination environment

filt_origin <- 0.3 # Niche filter/selection in origin
```

```
filt_movement <- 0.7 # Niche filter/selection in movement - will transition generation by generation; s
filt_destination <- 0.4 # Niche filter/selection in destination
```

Generate a list of filtering functions to pass to shuffle code - allows for gradual niche filter change AND shuffling (if using).

```
filt_movement_seq <- c(seq(filt_origin, filt_movement, length.out = ngen_movement*0.1), rep(filt_moveme
filtfun <- function(flt) {force(flt)
  fun2 <- function(x) {filt_gaussian(flt, 0.05, x)}
  return(fun2)}

filt_movement_lst <- vector("list", length(filt_movement_seq))
for (i in 1:length(filt_movement_seq)){
  filt_movement_lst[[i]] <- filtfun(filt_movement_seq[i])
}
```

Set the number of iterations/re-runs. 2 for demonstrative purposes.

```
n_iterations <- 2
```

Run simulation. Returns only iteration number as results are output directly to file.

```
lapply(1:n_iterations, FUN = function(iter){

#origin
initial <- lapply(1:ifelse(is.null(cohort_size), 1, cohort_size), function(x){origin_start[sample(nrow(
origin_section <- forward_shuffle(init_pools = initial, prob = prob_imm, prob.death = prob_death, gens =

#movement
movement_section <- forward_shuffle(init_pools = origin_section, prob = prob_imm, prob.death = prob_dea

#destination
destination_section <- forward_shuffle(init_pools = movement_section, prob = prob_imm, prob.death = prob
return(iter) #data are output to file
})
```

```
## [[1]]
## [1] 1
##
## [[2]]
## [1] 2
```

Read in and summarise simulation outputs for plotting.

```
fldrs <- paste0("output/", list.files(path = "output", pattern = "iteration"))

allRuns_summary <- do.call(rbind, lapply(1:length(fldrs), FUN = function(iter){
fldr <- fldrs[iter]

sub_fldrs <- list.files(fldr)[-1] #drop call.csv output file

do.call(rbind, lapply(1:length(sub_fldrs), function(sub_it){
pth <- paste0(fldr, "/", sub_fldrs[sub_it])

file_lst <- paste0(pth, "/", list.files(pth, pattern = "generation"))

splist <- lapply(file_lst, function(x){gsub("_sh", "", data.table::fread(x)$sp, perl = TRUE)}) #remove
```

```
data.frame(iter = iter, sub_it = sub_it, gen = 1:length(file_lst), SR =sapply(splist, function(x){
        SR <- length(unique(x))}, USE.NAMES = FALSE), Pevn = sapply(splist, function(x){
        tab <- table(x)
        Pi <- tab/sum(tab)
        Piel <- (-1 * (sum(Pi * log(Pi))))/log(length(tab))
        }, USE.NAMES = FALSE),
            medTrait = sapply(file_lst, function(x){median(data.table::fread(x)$trait)}),
            BrayCurtis = sapply(splist, function(x){

                first_data  <- table(splist[[1]])
                tab <- table(x)
                comb_data <- as.matrix(dplyr::bind_rows(first_data, tab))
                comb_data[is.na(comb_data)] <- 0

                return(1 - ((2*sum(matrixStats::colMins(comb_data))) / (sum(first_data) + sum(tab))))
                }))
}))

}))
```

Basic plotting of richness, evenness, median species trait, and Bray-Curtis Dissimilarity over time.

```
ggplot(data = allRuns_summary, aes(x = gen, y = SR))  + stat_summary(fun=min, geom="line", linetype = "d
```

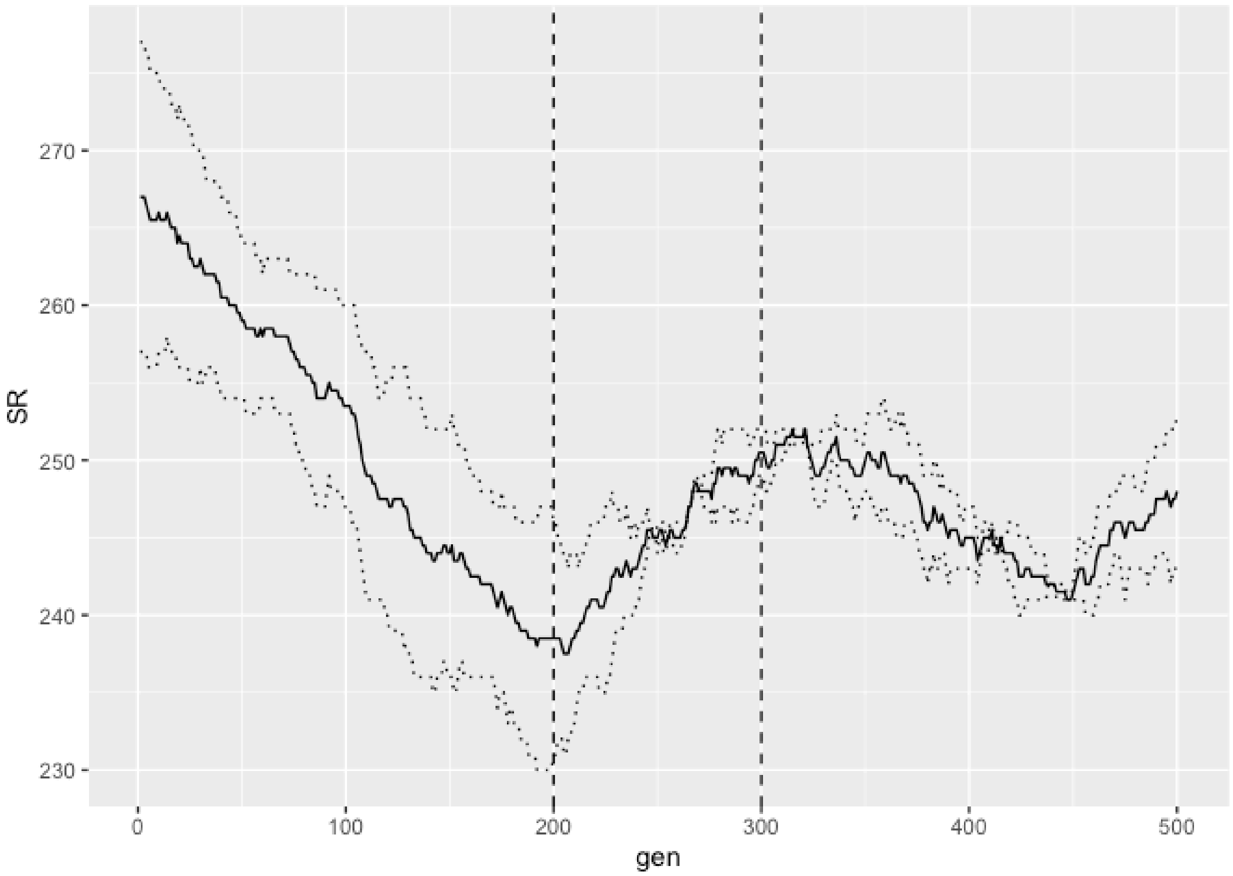

```
ggplot(data = allRuns_summary, aes(x = gen, y = Pevn)) + stat_summary(fun=min, geom="line", linetype =
```

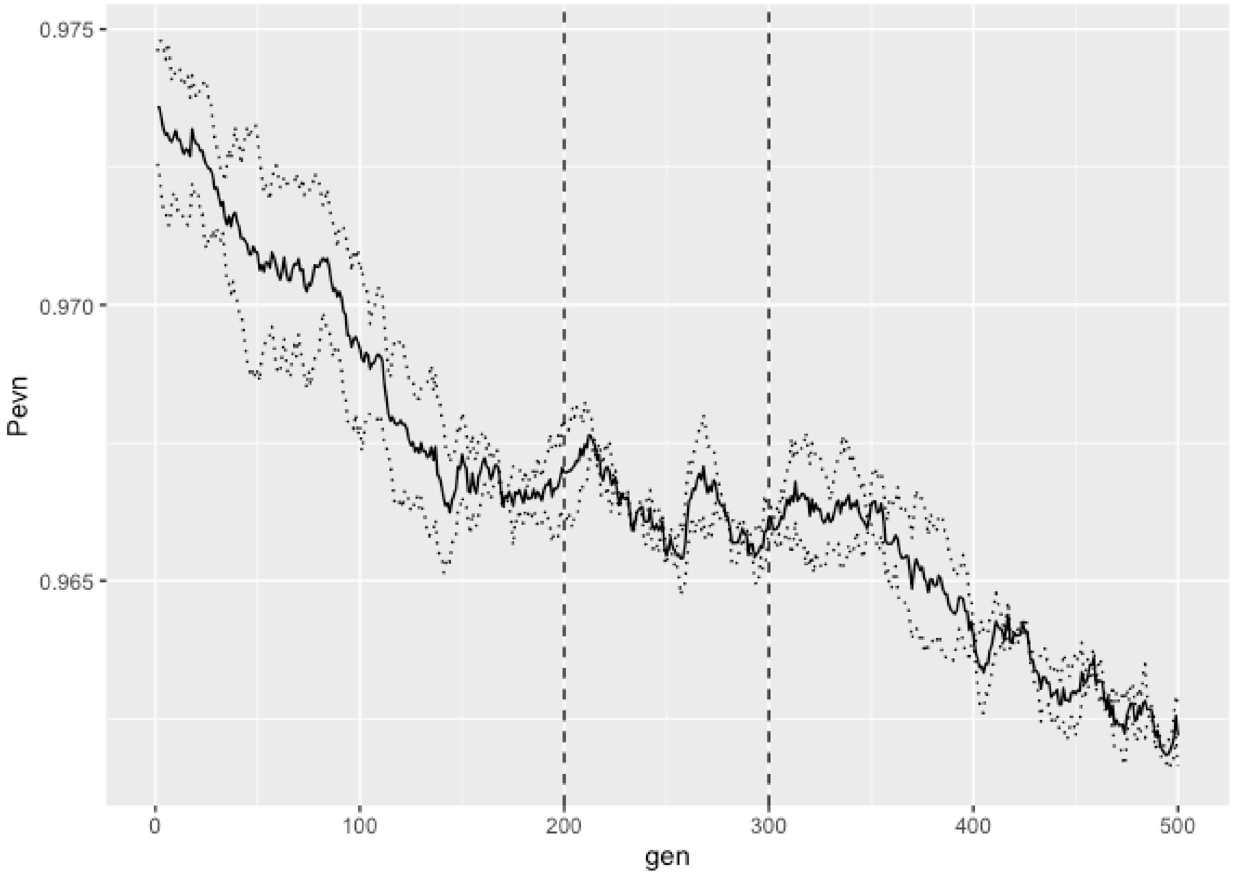


```
ggplot(data = allRuns_summary, aes(x = gen, y = medTrait)) + stat_summary(fun=min, geom="line", linetyp
```

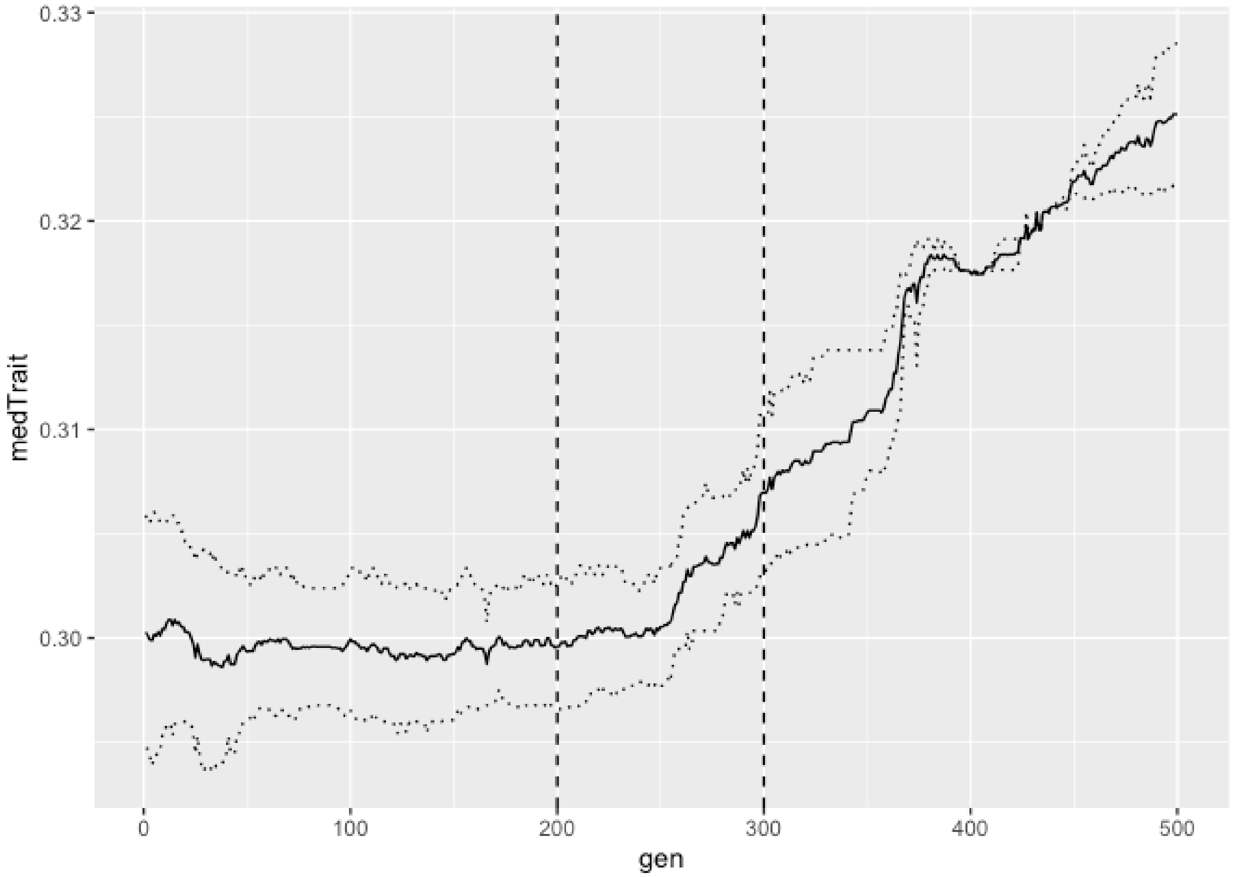


```
ggplot(data = allRuns_summary, aes(x = gen, y = BrayCurtis)) + stat_summary(fun=min, geom="line", linet
```

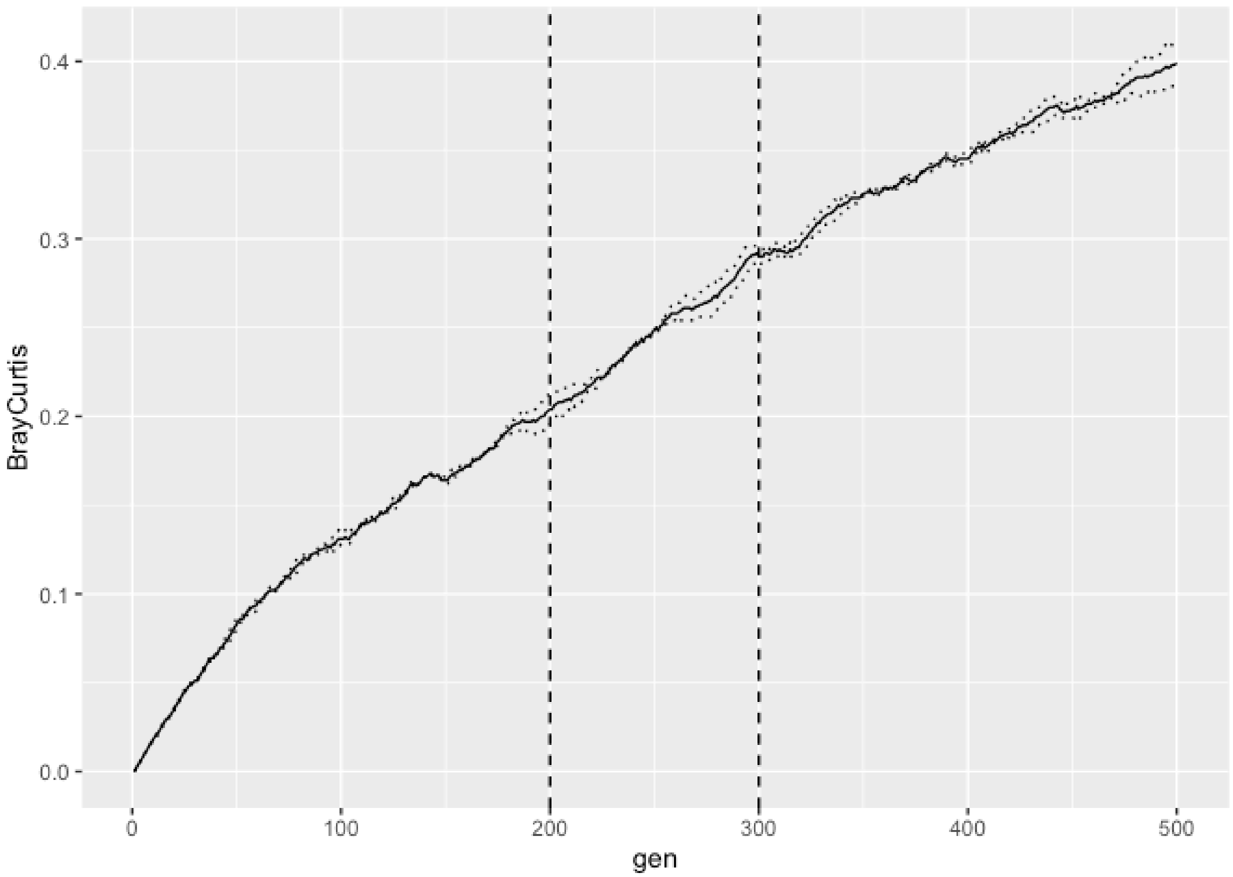

BrayCurtis
0.0
0.1
0.2
0.3
0.4
0
100
200
300
400
500
gen
