# Supplementary material for: Long-distance movement dynamics shape host microbiome richness and turnover
Source: FEMS Microbiol Ecol. 2024 Jun 10;100(7):fiae089. doi: 10.1093/femsec/fiae089 (PMC11212666; doi:10.1093/femsec/fiae089)
Supplement: fiae089_Supplemental_Files [file fiae089_supplemental_files.zip › supplementary data_revised.docx]

**Supplementary Material**


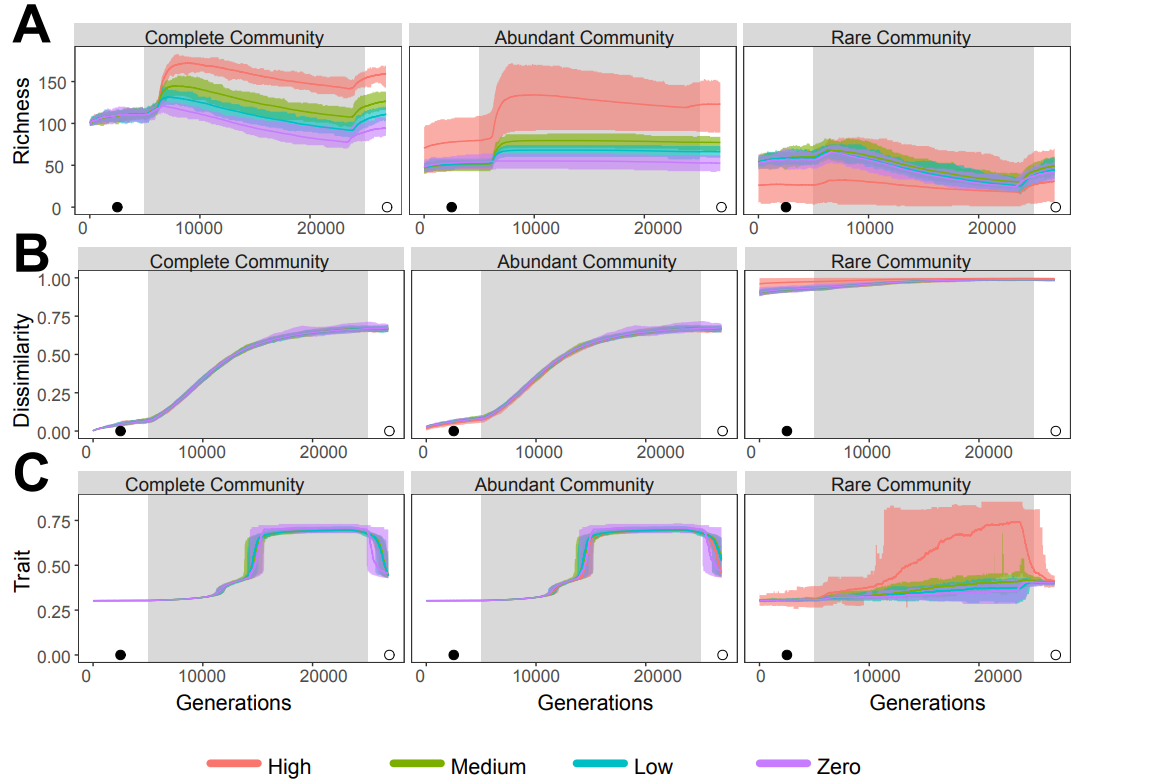


Supplementary Figure 1: Influence of environmental richness on the microbiome over the course of an extended migration in terms richness (A), Bray-Curtis dissimilarity to initial community (B), and the functional structure of the community based on median trait value (C). Community groups indicate the entire community, or the abundant sub-community (mean abundance of ≥0.01 over the entire simulation) or rare sub-community (mean abundance of < 0.01 over the entire simulation). Richness groups are relative and represent the richness of the environment during the migratory phase (in grey). Closed circle represents the ‘origin’ population and closed represents the ‘destination’ population.


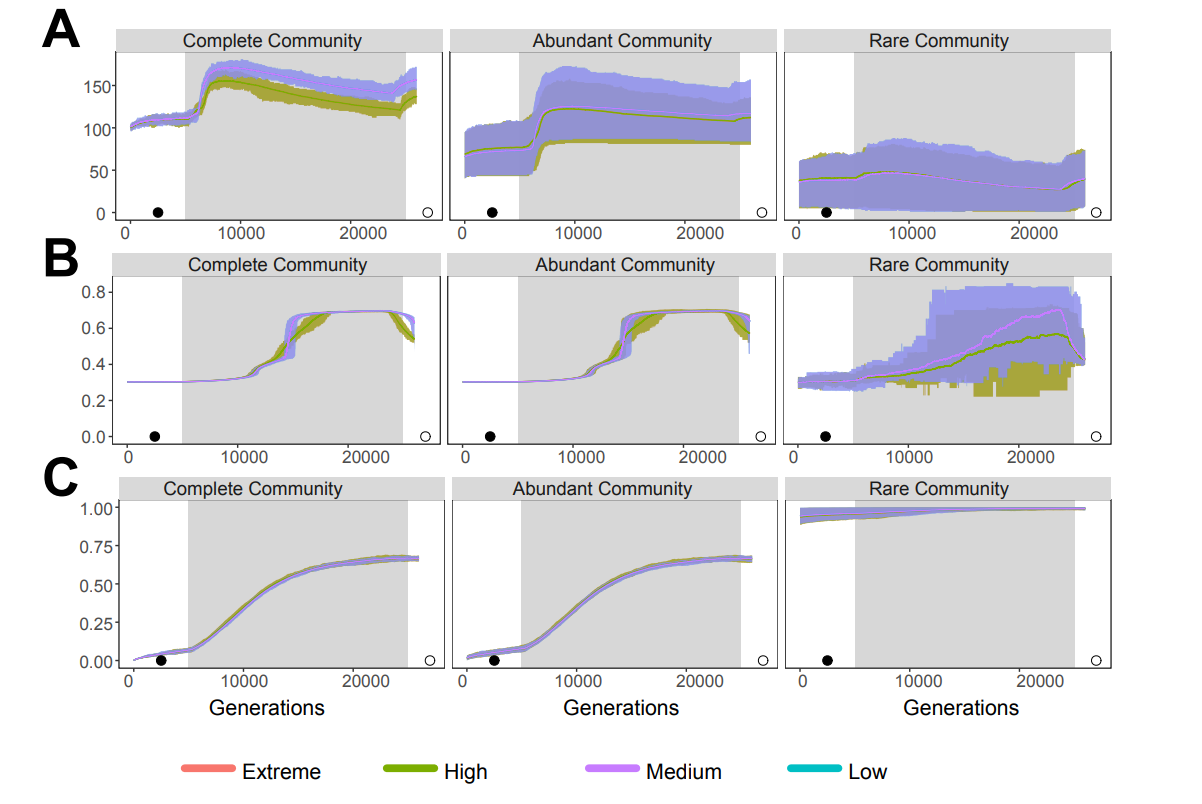


Supplementary Figure 2: Influence of selection strength on microbiota richness (A), Bray-Curtis dissimilarity to initial community (B), and the functional structure of the community based on median trait value (C). Community groups indicate the entire community, or the abundant sub-community (mean abundance of ≥0.01 over the entire simulation) or rare sub-community (mean abundance of < 0.01 over the entire simulation). Selection groups are based on a niche filter which changes in response to migration (in grey), note that “extreme” and “high” selection are identical, and “medium” and “low” selection are identical – resulting in only two groups being apparent. Closed circle represents the ‘origin’ population and closed represents the ‘destination’ population.


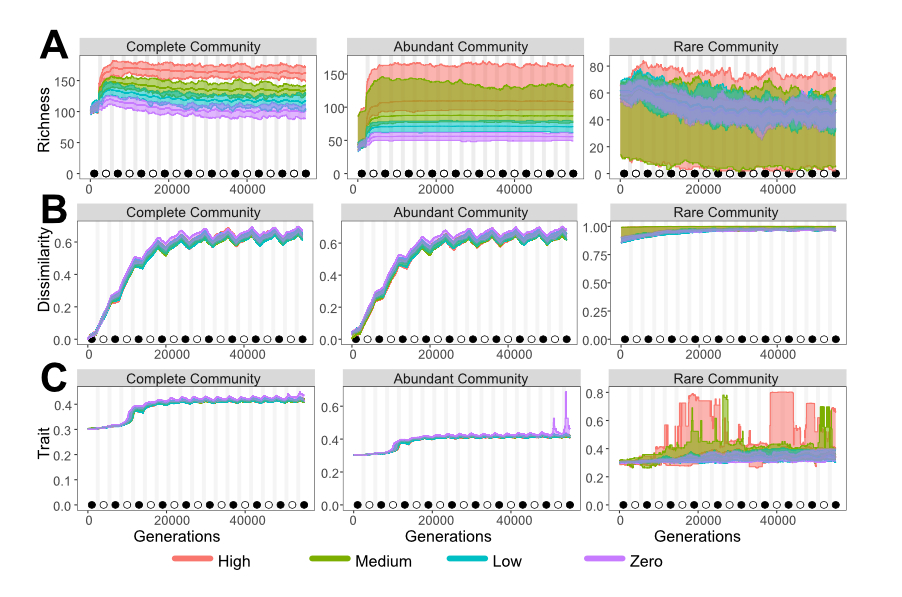


Supplementary Figure 3: Influence of environmental richness on the microbiota over the course of repeated migratory events in terms of richness (A), Bray-Curtis dissimilarity to initial community (B), and the functional structure of the community based on median trait value (C). Community groups indicate the entire community, or the abundant sub-community (mean abundance of ≥0.01 over the entire simulation) or rare sub-community (mean abundance of < 0.01 over the entire simulation). Richness groups are relative and represent the richness of the environment during the migratory phase (in grey). Closed circle represents the ‘origin’ population and closed represents the ‘destination’ population.

*Supplementary material*

**Simulation methods**

At each time step, every individual within the current community has a probability of dying, which is calculated as a function of the baseline death rate, the individual’s relative fitness in the current environment, and the degree of trait overlap (as a proxy for competition) with other species. As a ‘zero-sum’ simulation, dead individuals are immediately replaced by either ‘offspring’ of the remaining individuals or immigrants from the current environmental pool of species. The probability of an individual filling any empty slots is calculated as a function of that individual’s fitness. The resultant community is passed to the next time step of the simulation.


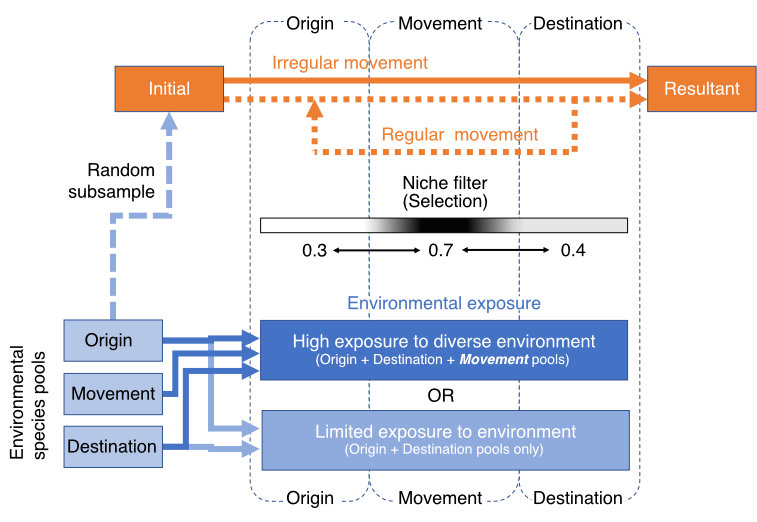


Supplementary Figure 4 Simulation work flow for creation of ecological simulations.

**Environmental species pools**

Three species pools were generated to represent the environmental microbiome of either the ‘origin’, ‘destination’, or ‘movement’ environments. Environmental pools comprised 10000 individuals which were assigned arbitrary species identities via sampling with replacement of species lists derived from either coastal (for origin and destination) or open ocean (for movement) water samples. These data were derived from water samples collected either as part of a coastal macroalgal sampling effort (Pearman *et al.* 2023), or off-shore sampling from the Munida Microbial Observatory Time Series dataset (Lockwood *et al.* 2022).

**Species traits and selection**

Species traits were pseudo-randomly assigned so that the most abundant species had traits that were close to the ideal trait value of their respective environments (i.e. assuming that the best adapted species were the most abundant), while species with lower abundance had traits that were increasingly more distant from the ideal trait of their environment. Where a species occurred in more than one environment, trait values were assigned in the environment in which the species was most abundant, and this value was used across all other environments. Once each species had been assigned a trait, random noise was added to the trait of each individual (-1/+1 bounded normal distribution, sigma = 0.02) so as to mimic natural within-species variability of traits.

**Simulation runs**

The initial host microbiome was generated using a random draw (without replacement) of 5000 individuals from the origin species pool (Zeng *et al.* 2015). The simulation was first run for 3000 generations under origin conditions to allow the microbiome to stabilise. The simulation was then run for another 2000 generations under origin conditions before transitioning to movement conditions, followed by a transition to destination conditions for 2000 generations. The length of time under movement conditions varied depending on the scenario; 1000 or 20 000 generations to simulate irregular movement. For the regular movement scenario this order was reversed and repeated etc. until a total of 50 000 generations had occurred (i.e., origin-movement-destination-movement-origin etc 2000/1000/2000/1000/2000 gens respectively). The environmental pool available for external recruitment was either a ‘Global’ pool, created by combining all three destination, origin, and movement pools or a ‘restricted’ pool, created by combining only the destination and origin pool. These were used for the high and low exposure simulations, respectively. For each scenario, 50 random iterations were performed.

At each time step, species richness was calculated as the total number of unique species in the current host microbiome. The median trait of all individuals in the current host microbiome was used as a proxy for community functional diversity. Bray-Curtis Dissimilarity, between the current host microbiome and the microbiome at generation 0 (i.e., after stabilisation at the origin), was calculated a measure of distance between the starting and current microbiomes. For each metric at each time step, the mean, standard deviation, and range was calculated across all 50 iterations.

**Model assumptions and justifications**

In line with recommendations that ecological theory and methods can be beneficially applied to microbial ecology (e.g. (Prosser *et al.* 2007; van den Berg *et al.* 2022)), our approach builds upon lessons learned in ecological modelling more broadly. Simulations integrate resource limitation and competition, niche requirements, and exchanges between the community and regional pools; in ecological modelling these three components have been identified as key drivers of observed patterns that are able to reproduce long-term ecological dynamics (e.g. (Ernest *et al.* 2008)). Owing to the lack of data around how microbiomes are affected by host movement, our simulations are necessarily simple and rely on a number of assumptions. As such, these simulations should be considered ‘demonstration’ models (*sensu* (Evans *et al.* 2013)):

*“Demonstration models are useful because they provide a toolkit for developing ecological theory… [but] they only provide elements of possible explanations of real systems. Demonstration models, at best, show that the modelled principles are sufficient to produce the phenomenon of interest; they do not help decide whether they are necessary. …Demonstrations do not need to be tested against specific data because they represent concepts rather than systems.”*

*Evans et al., (2013)*

This considers the maxim in ecological modelling that ‘simple models are more generalisable’ while acknowledging that more complex models are likely required to make explicit predictions for specific systems. Nevertheless, simple ‘demonstration’ models are useful to identify widespread phenomena (Evans *et al.* 2013) and relatively simple models can reproduce experimentally observed microecological patterns (Iii *et al.* 2019; Marsland, Cui and Mehta 2020), including those documented by the earth Microbiome and the Human Microbiome projects (Marsland *et al.* 2020). Furthermore, until more data, such as those from longitudinal studies or long-term time series (as commonly used in ecological modelling more broadly; e.g.(Ernest *et al.* 2008)), is available to parameterise more complex and/or specific microecological models, we considered simple demonstrative models the most appropriate approach. Simplified models carry the added benefit of being less computationally demanding. As more data become available, we recommend the use of more complex and specifically parameterised models.

*Specific assumptions and limitations:*

*i) Zero-sum model*

Throughout, the simulation runs as a ‘zero-sum’ model. That is, the overall community size starts at 5000 individuals and remains at 5000 individuals across all generations. Therefore, any individuals that die are immediately replaced by immigrants or offspring of incumbent species. This somewhat mirrors a real-world resource-limited community (the limiting resource here being space/the number of individual slots in the community), with resource limitation being identified as a key consideration in ecological modelling (Ernest *et al.* 2008) that is also “central to understanding both pattern and process in diverse microbial communities” (Mitri, Clarke and Foster 2016). Nevertheless, a zero-sum model cannot account for an unfilled space or underutilised resources, resource variation through time, or individuals of different species taking up different amounts of resources/space.

*ii) Niche continuum, fitness, and response*

The simulation assumes that all niche-related process occur on a univariate scale ranging from 0 to 1. This is a gross simplification of the multivariate niche space that species occupy but can be viewed as akin to distilling a multivariate niche down to its principal components, with the distance between a species or individual trait relative to the current environmental niche value acting as a simple representation of fitness in the current environment. The probability of an individual dying, immigrating, and/or mutating is calculated based on the distance between trait and environment and its interaction with baseline probabilities of each of these processes. Considering environments and niches on a univariate scale is also computationally efficient, with the addition of extra dimensions and their interactions with all other dimensions scaling computation requirements exponentially.

*iii) Species with overlapping traits compete directly*

The simulation assumes that species with similar trait values will directly compete with one another. While some degree of overlap is allowable, too much results in one species outcompeting the other, or neither species performing well. Competition between species is a key component of even relatively simple ecological models (Ernest *et al.* 2008), though interactions amongst microbes occupying the same niche space are likely more nuanced than this in practice (Prosser *et al.* 2007).

*iv) Origin and destination environments are more similar to each other than either is to the movement environment*

On the univariate niche continuum, the origin and destination environments are relatively close (0.3 and 0.4, respectively) while the movement environment is distanced from both (0.7). This is under the assumption that movement events start and end in similar locales (e.g., two coastal environments) and the movement occurs across an environment that is not suitable for long-term settlement (e.g., an open ocean environment). This is broadly in keeping with described movement and migration events, such is the case with movement of rafting invertebrates and some species of intertidal macroalgae (Fraser *et al.* 2022) as well as for whales - which despite passing through pelagic open oceanic waters, do not inhabit them continuously throughout the year.

*v) Transition between origin/destination* *and movement environments occurs gradually in the first 10% and last 10% of movement generations*

We assume that the environmental gradually changes over multiple generations (arbitrarily 10% of the movement time) rather than abruptly between one generation and the next. This is based on gradual environmental transitions between open ocean (i.e. movement) and coastal (i.e. origin/destination) environmental variables, such as sea surface temperature (Dlugosch *et al.* 2022), and observations that physiological changes in hosts are gradual rather than abrupt (Handby *et al.* 2022).

*vi) The community is exposed to a spatially homogenous environment*

At each generation, environmental selection is applied equally across all individuals of the community. Therefore, although traits (and subsequent responses) vary by species and individual, there is no allowance for microclimate variability across the microbiome. Heterogeneity in environment could be accommodated by dividing and then recombining the community at each time step in the simulation but this would add additional complexity (and computational overheads) to the model.

*vii) Within-species trait variability follows a normal distribution*

We added gaussian noise to species mean trait values to replicate within-species trait variability. Some species traits, such as thermal limits (Duffy *et al.* 2021) or cell volume (Wieczynski *et al.* 2021), are skew-normal or non-normally distributed among individuals. However, as environmental selection and traits represent a hypothetical trait that exist on a univariate scale in our simulations, and the direction and extent of skew varies considerably amongst species (Wieczynski *et al.* 2021), we opted to assume that within-species variability follows a normal distribution on average so as to represent the broadest possible range of trait-environment responses.

*viii) The most abundant species in environmental pools have traits closest to the ‘ideal’ trait for the respective environment*

When setting up environmental species pools for origin, destination, and movement environments, traits were assigned to each species. We assumed that the most abundant species in each pool was also the best adapted species in its environment and would, therefore, have a trait value equal to or very close to the environmental value. Under the assumption that the univariate trait/niche continuum represents relative fitness, this assumption is intuitive and is based on evidence with the literature (Wan *et al.* 2021; Liu *et al.* 2023). Nevertheless, the nuances of microbial interaction and competition (Prosser *et al.* 2007) may mean that species with less than ideal traits are most abundant in ‘real world’ communities.

**References**

van den Berg NI, Machado D, Santos S *et al.* Ecological modelling approaches for predicting emergent properties in microbial communities. *Nat Ecol Evol* 2022;**6**:855–65.

Dlugosch L, Poehlein A, Wemheuer B *et al.* Significance of gene variants for the functional biogeography of the near-surface Atlantic Ocean microbiome. *Nat Commun* 2022;**13**:456.

Duffy GA, Kuyucu AC, Hoskins JL *et al.* Adequate sample sizes for improved accuracy of thermal trait estimates. *Funct Ecol* 2021;**35**:2647–62.

Ernest SKM, Brown JH, Thibault KM *et al.* Zero Sum, the Niche, and Metacommunities: Long‐Term Dynamics of Community Assembly. *Am Nat* 2008;**172**:E257–69.

Evans MR, Grimm V, Johst K *et al.* Do simple models lead to generality in ecology? *Trends Ecol Evol* 2013;**28**:578–83.

Fraser CI, Dutoit L, Morrison AK *et al.* Southern Hemisphere coasts are biologically connected by frequent, long-distance rafting events. *Curr Biol* 2022;**32**:3154-3160.e3.

Handby T, Slezacek J, Lupi S *et al.* Changes in Behaviour and Proxies of Physiology Suggest Individual Variation in the Building of Migratory Phenotypes in Preparation for Long-Distance Flights. *Front Ecol Evol* 2022;**10**.

Iii RM, Cui W, Goldford J *et al.* Available energy fluxes drive a transition in the diversity, stability, and functional structure of microbial communities. *PLOS Comput Biol* 2019;**15**:e1006793.

Liu X, Li H, Song W *et al.* Distinct ecological mechanisms drive the spatial scaling of abundant and rare microbial taxa in a coastal sediment. *J Biogeogr* 2023;**50**:909–19.

Lockwood S, Greening C, Baltar F *et al.* Global and seasonal variation of marine phosphonate metabolism. *ISME J* 2022;**16**:2198–212.

Marsland R, Cui W, Goldford J *et al.* The Community Simulator: A Python package for microbial ecology. *PLOS ONE* 2020;**15**:e0230430.

Marsland R, Cui W, Mehta P. A minimal model for microbial biodiversity can reproduce experimentally observed ecological patterns. *Sci Rep* 2020;**10**:3308.

Mitri S, Clarke E, Foster KR. Resource limitation drives spatial organization in microbial groups. *ISME J* 2016;**10**:1471–82.

Munoz F, Grenié M, Denelle P *et al.* ecolottery: Simulating and assessing community assembly with environmental filtering and neutral dynamics in R. *Methods Ecol Evol* 2018;**9**:693–703.

Pearman WS, Morales SE, Vaux F *et al.* Differences in density: taxonomic but not functional diversity in seaweed microbiomes affected by an earthquake. 2023:2023.02.08.527737.

Prosser JI, Bohannan BJM, Curtis TP *et al.* The role of ecological theory in microbial ecology. *Nat Rev Microbiol* 2007;**5**:384–92.

R Core Team. R: A language and environment for statistical computing. 2022.

Wan W, Gadd GM, Yang Y *et al.* Environmental adaptation is stronger for abundant rather than rare microorganisms in wetland soils from the Qinghai-Tibet Plateau. *Mol Ecol* 2021;**30**:2390–403.

Wieczynski DJ, Singla P, Doan A *et al.* Linking species traits and demography to explain complex temperature responses across levels of organization. *Proc Natl Acad Sci* 2021;**118**:e2104863118.

Zeng Q, Sukumaran J, Wu S *et al.* Neutral models of microbiome evolution. *PLOS Comput Biol* 2015;**11**:e1004365.
