# Supplementary figures and images for: Long-distance movement dynamics shape host microbiome richness and turnover
Source: FEMS Microbiol Ecol. 2024 Jun 10;100(7):fiae089. doi: 10.1093/femsec/fiae089 (PMC11212666; doi:10.1093/femsec/fiae089)

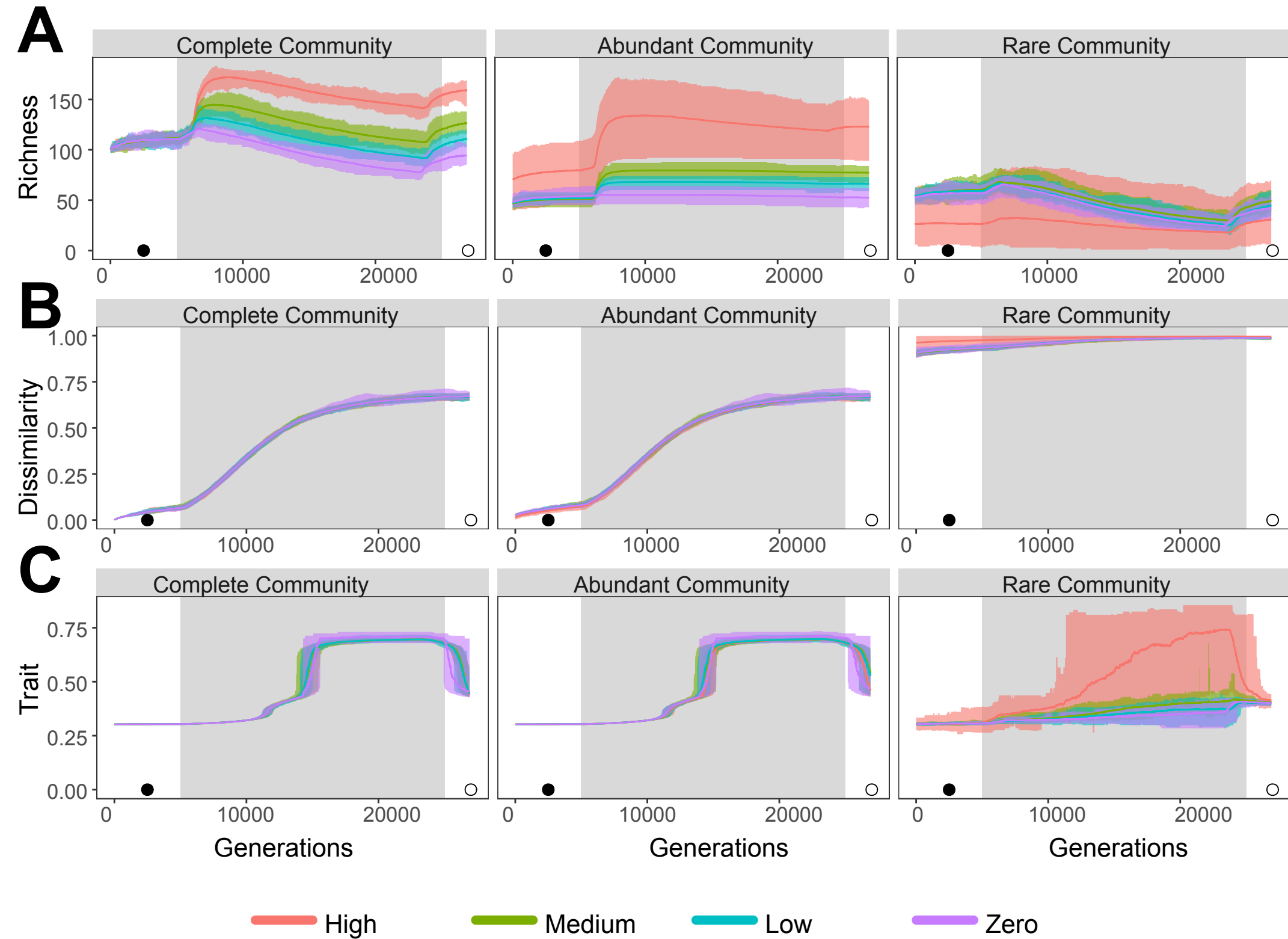

Supplement: fiae089_Supplemental_Files [file fiae089_supplemental_files.zip › supp data fig1.pdf]

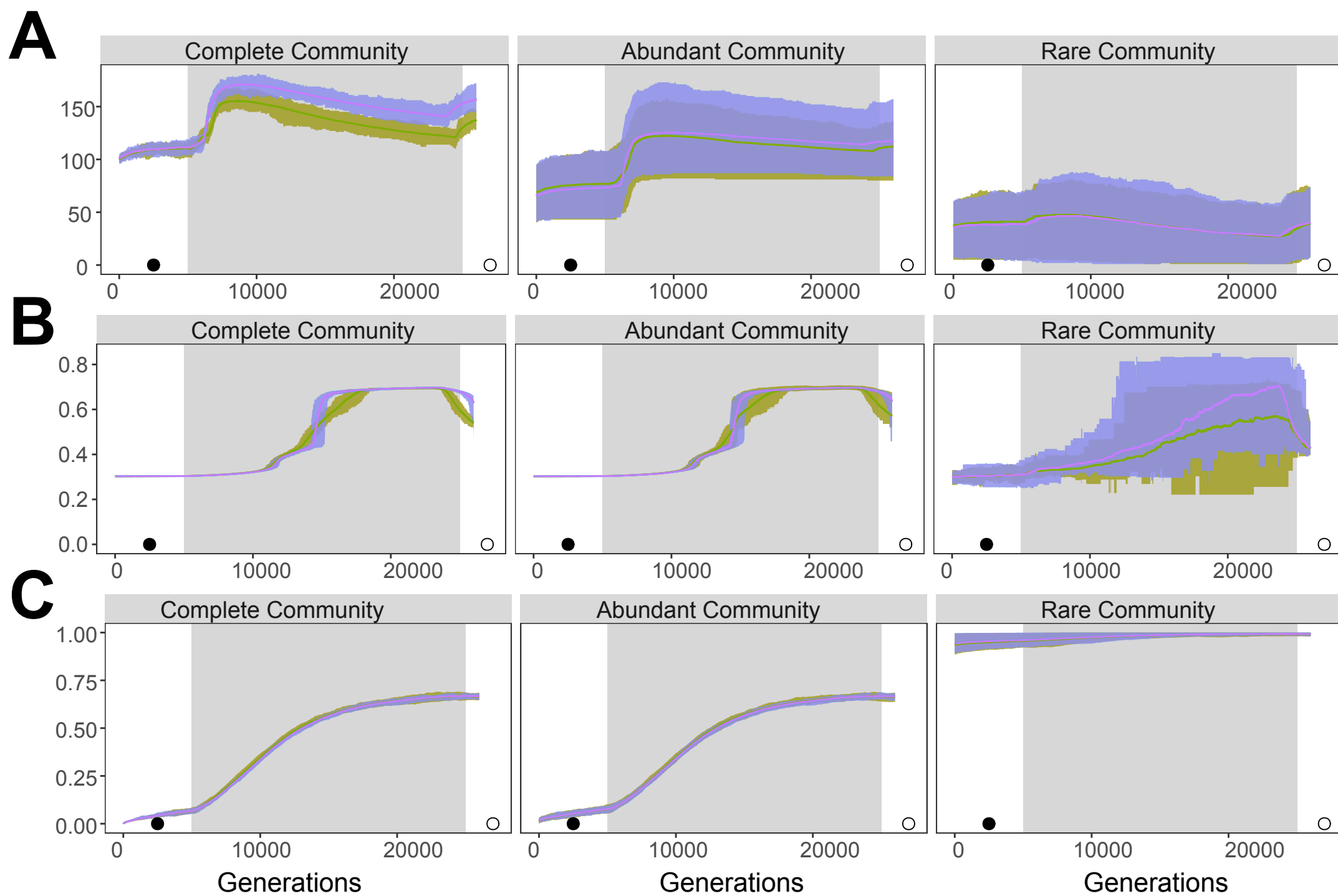

Supplement: fiae089_Supplemental_Files [file fiae089_supplemental_files.zip › supp data fig2.pdf]

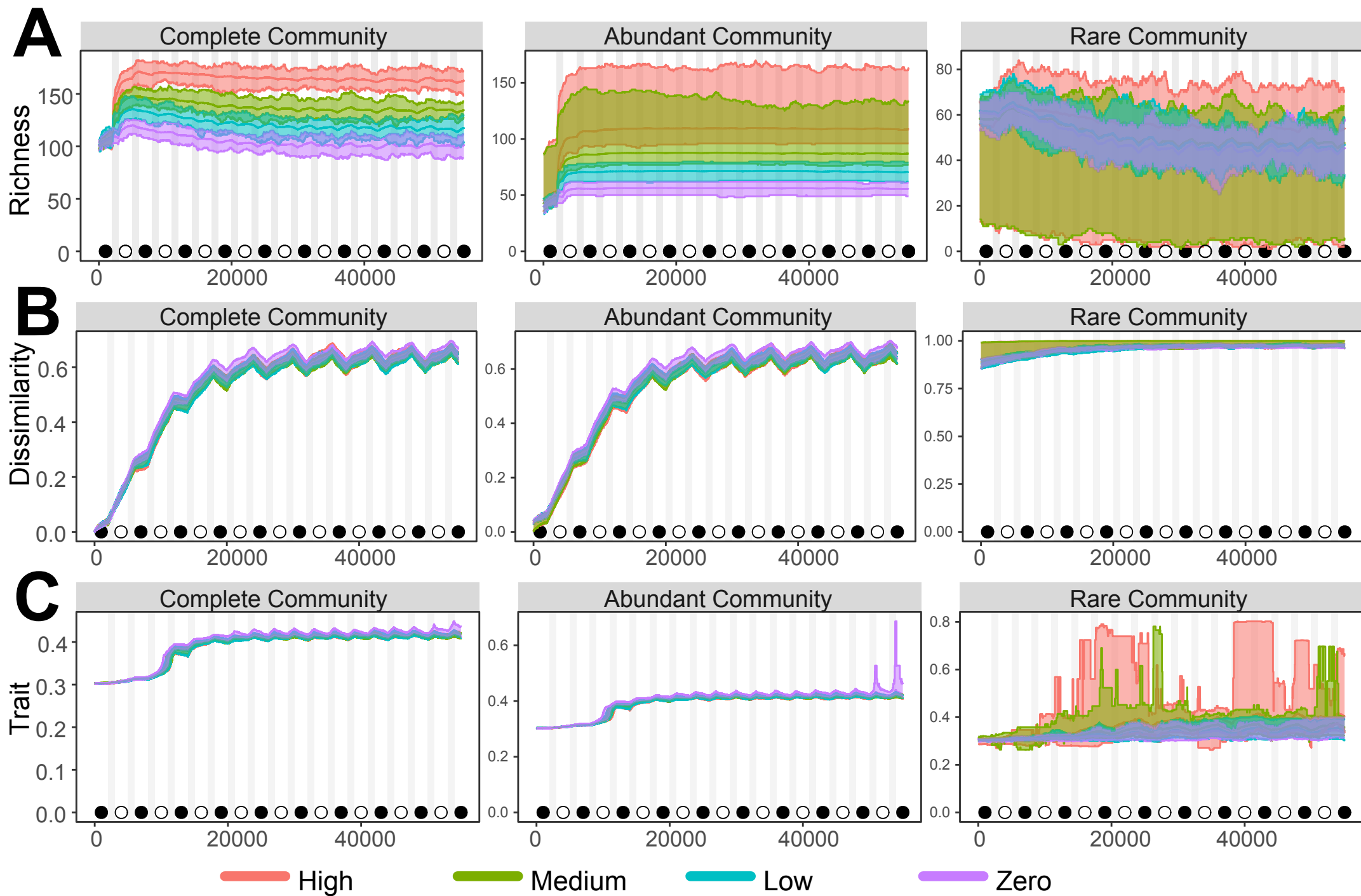

Supplement: fiae089_Supplemental_Files [file fiae089_supplemental_files.zip › supp data fig3.pdf]

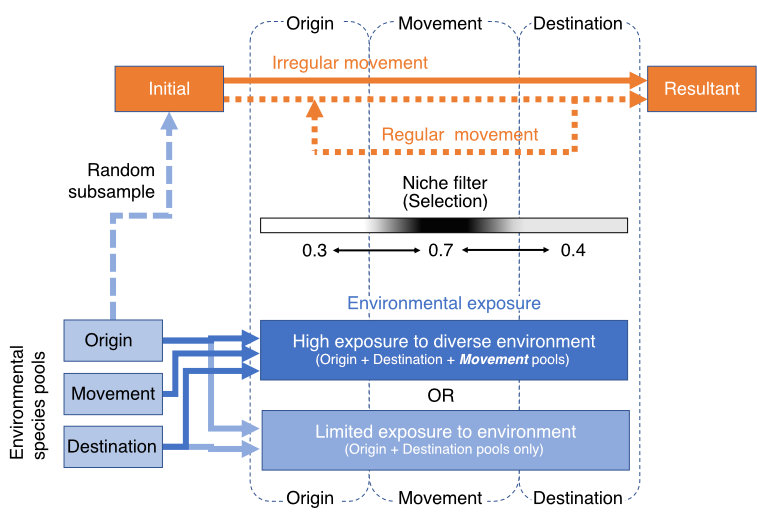

Supplement: fiae089_Supplemental_Files [file fiae089_supplemental_files.zip › supp data fig4_flow.jpg]
